# Ectopic breast tissue with milky secretions on the axillae in a lactating Filipino female: A case report

**DOI:** 10.1016/j.jdcr.2025.06.004

**Published:** 2025-06-18

**Authors:** Alyssa Felsophie S. Silor, Marian Rosel D. Villaverde, Claudine Yap Silva

**Affiliations:** Department of Dermatology, University of the Philippines – Philippine General Hospital, Manila, Philippines

**Keywords:** accessory breasts, axillae, case report, ectopic breast tissue, mammary gland, polymastia, supernumerary breast tissue

## Introduction

Breast tissue found in areas other than the anterior aspect of the thoracic wall or pectoral region is termed ectopic or accessory breast tissue.^1^^,^^2^ The embryonic mammary ridge or milk line refers to the mammary streaks that develop along the ventral surface from the axilla to the groin, which eventually regresses during embryologic development except on the anterior aspect of the thoracic wall.^1^ Lack of regression entails the formation of an ectopic or accessory breast tissue.

Although it is already present at birth, ectopic breast tissue stays dormant until there are hormonal changes.^3^ It is a rare condition found in 2% to 6% of females and 1% to 3% of males and is commonly observed in women during puberty, pregnancy, and lactation periods.^1^, ^2^, ^3^ It is usually sporadic but may be familial in 6% of cases.^2^

## Case report

A 35-year-old Filipino female, gravida 4 para 4, presented with a 15-year history of recurrent swelling of bilateral axillary regions, with milky secretions from the overlying hair follicles. This initially presented during the immediate postpartum period of her first pregnancy and was noted to recur with each succeeding pregnancy with spontaneous resolution after weaning from breastfeeding. No changes in the skin such as redness, warmth, discharge, bleeding, tenderness, or pruritus were noted. A review of the systems was unremarkable. The patient has no known comorbidities, and there were no similar lesions in the family. She is a nonsmoker and occasional alcoholic beverage drinker. She has had regular menses since 13 years old with no noted cyclical swelling of the axillae.

**Fig 1:**
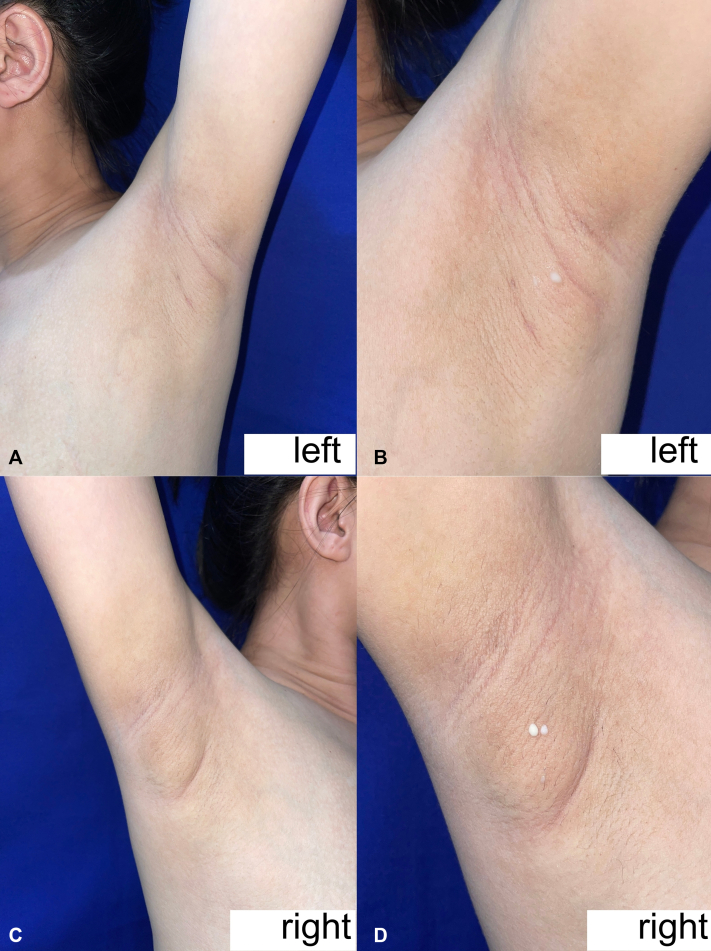
**A-D,** Multiple ill-defined, irregularly shaped, soft, skin-colored, nontender masses with milky secretions on the bilateral axillary areas. Note the absence of a nipple-areolar complex on the overlying skin.

A focused dermatologic examination revealed no evident cutaneous lesions aside from ill-defined, irregularly shaped, soft, skin-colored, nontender masses on the bilateral axillary areas measuring approximately 5.5 × 4.2 cm on the right axilla and 3.9 × 0.9 cm on the left axilla (Fig 1, *A*, *B*). It lacked an associated nipple-areola complex and was classified as class 4 polymastia. There were milky secretions from the hair follicles upon manual expression (Fig 1, *C*, *D*). No cervical and axillary lymphadenopathies were palpated. Breast examination was unremarkable save for the breast engorgement secondary to lactation. The primary working impression was accessory breasts on the bilateral axillae.

**Fig 2:**
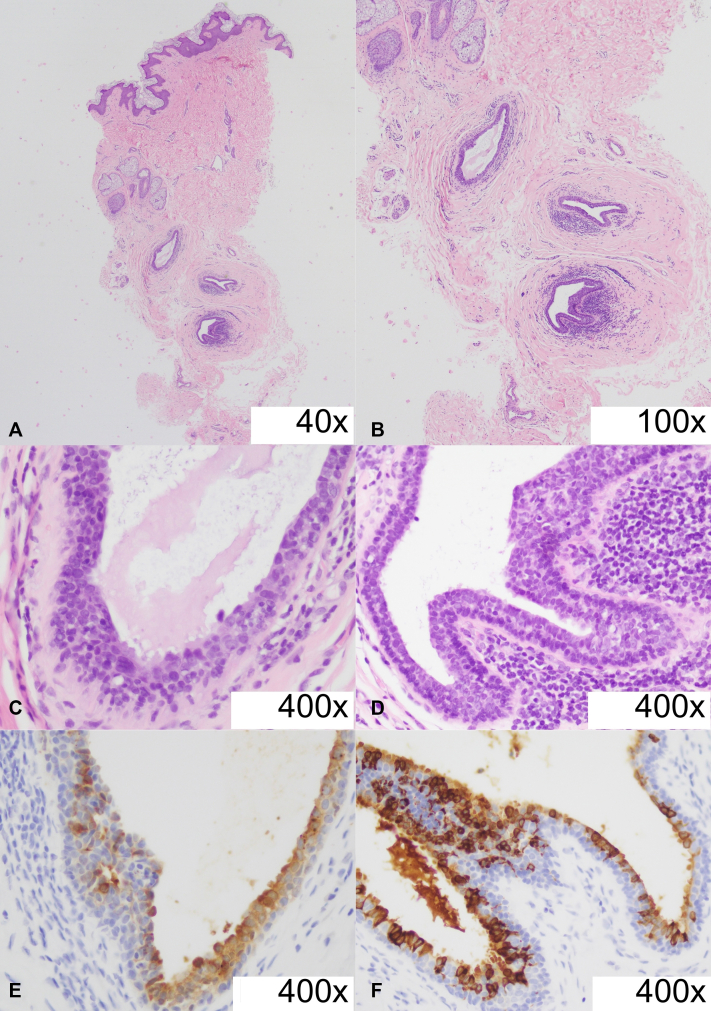
Photomicrograph showing several glandular structures in the reticular dermis, with a hyperplastic epithelium composed of cuboidal to columnar cells with decapitation secretion, and an outer discontinuous layer of spindle-shaped cells (hematoxylin-eosin stain: **A,** 40×, **B,** 100×, **C,** 400×, and **D,** 400×). Immunohistochemical stain showed strong cytoplasmic and membranous staining of glandular cells (mammaglobin stain: **E,** 400× and **F,** 400×).

The punch biopsy of a hair follicle revealed ectopic glandular epithelium. There was noted basketweave orthokeratosis, papillomatosis, and mild acanthosis on the epidermis. There were several glandular structures in the reticular dermis, with a hyperplastic epithelium composed of cuboidal to columnar cells with decapitation secretions, and an outer discontinuous layer of spindle-shaped cells (Fig 2, *A*-*D*). There were dense lymphohistiocytic inflammatory infiltrates surrounding the glandular structures. The biopsy findings were consistent with an ectopic breast tissue, which was supported by a strong cytoplasmic and membranous mammaglobin staining of glandular cells (Fig 2, *E*, *F*).

**Fig 3:**
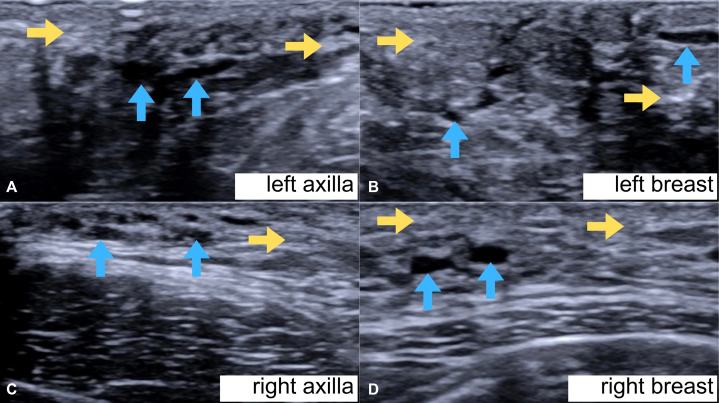
Focused ultrasound of bilateral axillae **(A**, **C)** and breasts **(B**, **D)**. *Yellow arrows* point to fibroglandular parenchyma while *blue arrows* show dilated ducts.

Ultrasound of the axillae showed bilateral accessory breast tissues with prominent duct ectasia with intraductal contents, whereas the breast ultrasound was unremarkable (Fig 3).

The patient is not desirous of excision due to the expectation of breast tissue regression upon cessation of breastfeeding. She was advised regular follow-ups for monitoring alongside regular breast cancer screening.

## Discussion

Axillary breast tissue can occur unilaterally or bilaterally and is often not associated with a distinct areola or nipple.^4^ In the case presented, the patient was only able to observe the ectopic breast tissue postpartum during breastfeeding in which she noticed milk-like fluid from the hair follicles in the axillae, similar to some case reports.^5^ Engorgement and associated symptoms become worse with subsequent pregnancies.^4^ Its presentation is correlated with hormonal changes.^1^ These may have gone unnoticed by the patient during childhood because she was asymptomatic. Due to the rarity of this condition and the absence of a nipple-areolar complex particularly in this case, these masses may appear nonmammary in origin. Patients have been misdiagnosed with lipoma, follicular cysts, lymphadenopathy, and hidradenitis suppurativa.^6^

There are a few reports of accessory axillary breast tissue in adults, but even fewer are noted in the pediatric population. A retrospective study focusing on adolescents noted its association with cyclic pain and menstruation, and upon excision, no recurrences were reported.^7^

Ectopic breast tissue can undergo similar pathological changes in a normal breast like mastitis, fibrocystic disease, and fibroadenoma.^3^ One reported case had fibrocystic changes in bilateral ectopic breast tissue in the axillae, which was successfully excised with no recurrences.^2^ There are a few studies suggesting that ectopic breast tissue might have a higher risk of malignant degeneration hence the need to consider this condition and work-up the patient for early detection.^6^

Imaging modalities that may be utilized in these cases include ultrasound, mammography, and magnetic resonance imaging.^2^ Bilateral ultrasonography is the recommended imaging for preoperative diagnosis which will show breast tissue that is indistinguishable from the regular breast where fibroductal tissue and fat lobules are visualized.^7^^,^^8^ Magnetic resonance imaging is only indicated in atypical cases, but this is rarely done.^8^

Definitive diagnosis can be obtained by histopathologic examination through fine needle aspiration or punch biopsy, which should show similar histologic findings with a normal breast tissue.^1^ Microscopic examination may show mature adipose tissue interspersed with breast tissue with a lobular architecture.^3^ The ducts and acini are lined by ductular epithelial cells. Immunohistochemical stains support the histopathologic diagnosis, and in 1 case, estrogen and progesterone receptors (ER and PR) stained the glandular epithelium.^9^ Another report on axillary breast tissue used estrogen receptor, progesterone receptor, C-erbB2, GCDPF-15, and mammaglobin as immunomarkers.^10^

Surgical excision is suggested in cases where lesions are symptomatic, show suspicious features on biopsy, or are a cause of cosmetic concern.^3^ Otherwise, watchful waiting and observation may be done, as with this case. Symptoms to watch out for include firm to hard, fixed masses with retraction.^5^ Despite possible malignant transformation, prophylactic surgical excision is not recommended.^1^

Ectopic breast tissue is an uncommon entity, which may be considered in the differential diagnoses of patients presenting with unilateral or bilateral axillary masses. It is usually evident during hormonal changes like puberty, pregnancy, and lactation. Proper work-up is prudent due to its potential for developing benign and malignant breast conditions. Management is conservative unless symptomatic and with cosmetic concerns.

## Conflicts of interest

None disclosed.
